# Serum Angiopoietin-Like Protein 2 Concentrations Are Independently Associated with Heart Failure

**DOI:** 10.1371/journal.pone.0138678

**Published:** 2015-09-23

**Authors:** Chi-Lun Huang, Yen-Wen Wu, Chih-Cheng Wu, Juey-Jen Hwang, Wei-Shiung Yang

**Affiliations:** 1 Department of Internal Medicine, Taoyuan General Hospital, Ministry of Health and Welfare, Taoyuan, Taiwan; 2 Department of Internal Medicine, National Taiwan University Hospital, Taipei, Taiwan; 3 Department of Nuclear Medicine, National Taiwan University Hospital, Taipei, Taiwan; 4 Department of Nuclear Medicine and Cardiovascular Medical Center (Cardiology), Far Eastern Memorial Hospital, New Taipei City, Taiwan; 5 National Yang-Ming University School of Medicine, Taipei, Taiwan; 6 Department of Internal Medicine, National Taiwan University Hospital Hsin-Chu branch, Hsinchu City, Taiwan; 7 Graduate Institute of Clinical Medicine, College of Medicine, National Taiwan University, Taipei, Taiwan; East Tennessee State University, UNITED STATES

## Abstract

**Objective:**

Angiopoietin-like protein 2 (ANGPTL2), which is mainly expressed from adipose tissue, is demonstrated to be involved in obesity, metabolic syndrome, and atherosclerosis. Because several adipocytokines are known to be associated with heart failure (HF), here we investigated the association of ANGPTL2 and HF in Taiwanese subjects.

**Methods and Results:**

A total of 170 symptomatic HF patients and 130 age- and sex-matched controls were enrolled from clinic. The echocardiography was analyzed in each patient, and stress myocardial perfusion study was performed for clinical suspicion of coronary artery disease. Detailed demographic information, medications, and biochemical data were recorded. Circulating adipocytokines, including tumor necrosis factor-alpha (TNF-α), adiponectin, adipocyte fatty acid-binding protein (A-FABP) and ANGPTL2, were analyzed. Compared with the control group subjects, serum ANGPTL2 concentrations were significantly higher in HF group patients. In correlation analyses, ANGPTL2 level was positively correlated to creatinine, fasting glucose, triglyceride, hsCRP, TNF-α, NT-proBNP and A-FABP levels, and negatively correlated with HDL-C and left ventricular ejection fraction. In multiple regression analysis, A-FABP, hsCRP, and HDL-C levels remained as independent predictors for ANGPTL2 level. To determine the association between serum ANGPTL2 concentrations and HF, multivariate logistic regression analyses were performed with subjects divided into tertiles by ANGPTL2 levels. For the subjects with ANGPTL2 levels in the highest tertile, their risk of HF was about 2.97 fold (95% CI = 1.24–7.08, P = 0.01) higher than those in the lowest tertile.

**Conclusion:**

Our results demonstrate a higher circulating ANGPTL2 level in patients with HF, and the upregulating ANGPTL2 levels might be associated with metabolic derangements and inflammation.

## Introduction

Heart failure (HF), a growing cause of morbidity and mortality, represents a major global health problem [1]. This complex clinical syndrome can result from numerous structural/functional cardiac disorders such as coronary artery disease (CAD), hypertensive heart disease, myocardial/pericardial disease, or valvular heart disease [2]. Apart from the associated hemodynamic changes, the neurohormonal and metabolic abnormalities associated with HF, including elevated circulating catabolic steroids, catecholamines, pro-inflammatory cytokines, and growth hormone, have received increased attention [3]. These abnormalities lead to progressive catabolism, weight loss, and cachexia [4]. Adipose tissue dysfunction with excessive adipocytokines production was also demonstrated to be involved in the development of HF and related to cardiometabolic complications via insulin resistance and chronic inflammation [5,6].

Angiopoietin-like protein 2 (ANGPTL2), as one of the eight members of the ANGPTL family, is involved in angiogenesis and tissue repair [7]. However, ANGPTL2 overexpression can cause chronic inflammation and subsequent irreversible pathological tissue remodeling, and is associated with obesity, metabolic disease, type 2 diabetes, atherosclerosis, and possibly some cancers [8]. In mice and human studies, ANGPTL2 is abundantly expressed in adipose tissues as a key mediator that links obesity, adipose tissue inflammation, and systemic insulin resistance [9,10]. An association with cardiovascular disease (CVD) was reported, as the perivascular adipose tissue-secreted ANGPTL2 accelerates both the vascular inflammation and pathological vascular tissue remodeling [11]. In seniors, serum ANGPTL2 levels positively correlated with both the intimal-medial thickness and the presence of arterial plaques [12]. Abundant ANGPTL2 expression was also observed in the atheromatous plaques of CAD patients, particularly in endothelial cells and infiltrated macrophages [12]. Recent studies demonstrated higher ANGPTL2 plasma levels in CAD and acute coronary syndrome patients than in healthy subjects, thereby correlating with disease severity [13,14]. In subjects with diabetes, serum ANGPTL2 levels were associated with carotid intima-media thickness [15]. However, the evidence of the association between ANGPTL2, cardiac function, and HF is currently lacking. Therefore, this study aimed to investigate whether circulating ANGPTL2 levels are associated with HF.

## Materials and Methods

### Ethics statement

This study was approved by the institutional review board of Taoyuan General Hospital. Written informed consent was obtained from each patient before enrollment. Medical records and patient information were anonymized and de-identified prior to analysis.

### Study design

This cross-sectional study enrolled 170 symptomatic HF patients (126 men; mean age, 67 years). All of the patients attended Taoyuan General Hospital and National Taiwan University Hospital, Hsin-Chu branch, between July 2010 and June 2012. Eligible patients had chronic stable HF diagnosed by an individual physician for at least 6 months. HF was diagnosed according to the American College of Cardiology/American Heart Association 2005 Guideline Update for the Diagnosis and Management of HF [16]. Patients who had evidence of acute inflammatory or infectious disease, decompensated liver disease, end-stage renal disease, active malignancy, acute coronary syndrome, or a stroke within 3 months prior to the investigation were excluded. Control subjects (n = 130), who had no identifiable HF symptoms, were selected from cardiovascular clinics and matched by age and sex. None of our participant took thiazolidinediones, which have been known to regulate the expression of ANGPTL2 [12].

Demographic information, including height, weight, cardiovascular risk factors, comorbid conditions, and list of current medications, was obtained from the medical records of the patients. M-mode and two-dimensional echocardiography were performed in all of the participants. The left atrial dimension (LAd), left ventricular end-diastolic dimension (LVEDd), left ventricular end-systolic dimension (LVESd), and left ventricular ejection fraction (LVEF) were evaluated and recorded in a blinded manner. HF with normal or mildly abnormal LVEF (≥50%) was defined as HF with a preserved ejection fraction (HFpEF). LVEF <50% was defined as HF with a reduced LVEF (HFrEF) [17].

As previously described, Tl-201 dipyridamole single-photon emission computed tomography (SPECT) was performed in 167 subjects (93 in the HF group and 74 in the control group) for the clinical suspicion of ischemic cardiomyopathy [18]. The regional myocardial uptake was normalized and assessed by using the 17-segment model and a semiquantitative scoring system for defect severity as recommended by the American Heart Society of Nuclear Cardiology [19]. The summed scores were calculated from the segmental scores, including a summed rest score (SRS; sum of the 17 segmental rest scores) and a summed stress score (SSS; sum of the 17 segmental stress scores). Scan findings were considered normal if the SSS was <4, mildly to moderately abnormal if the SSS was between 5 and 12, and severely abnormal if the SSS was >12 [20]. Ischemic cardiomyopathy was defined as >50% stenosis on coronary angiography, a history of myocardial infarction or coronary intervention, a history of chest pain with pathological Q waves on electrocardiography, or SSS >4 in the SPECT analysis.

The laboratory examinations included renal function tests, as well as tests for fasting glucose, lipid profile, high-sensitivity C-reactive protein (hsCRP), N-terminal pro-brain natriuretic peptide (NT-proBNP), tumor necrosis factor-alpha (TNF-α), adiponectin, adipocyte fatty acid-binding protein (A-FABP), and ANGPTL2 levels. The estimated glomerular filtration rate (eGFR) was calculated by the Modification of Diet in Renal Disease (MDRD) equation: 186 x (creatinine / 88.4)^-1.154^ x (age)^-0.203^ x (0.742 if female). Serum ANGPTL2 (Immuno-Biological Laboratories Co., Ltd, Gunma, Japan), A-FABP (BioVendor Laboratory Medicine, Inc. Brno, Czech Republic), TNF-α (R&D Systems, Minneapolis, MN, USA), and adiponectin (B-Bridge International, Inc. Cupertino, CA, USA) concentrations were then analyzed by using the enzyme-linked immunosorbent assay method, according to the manufacturers’ instructions.

### Statistical analysis

Data were reported as mean ± standard deviation values for normal distributions or as median values with interquartile ranges for skewed variables. Group comparisons were performed by using a two-sample *t*-test in normally distributed variables, and a Mann-Whitney U-test in variables not normally distributed. The univariate relationships between ANGPTL2 and clinical variables, the serum biomarkers, echocardiographically derived parameters, and SPECT findings were assessed by using the Spearman correlation coefficient (rho). After logarithmic transformation, a multiple forward stepwise regression analysis, which included all significant variables in Spearman correlation analysis, was then performed to identify independent parameters that correlated with ANGPTL2 level. In order to determine the independent HF predictors, multivariate logistic regression analyses were performed with the ANGPTL2 levels divided into tertiles. The analyses were performed by using the Stata statistical software (Version 10.0, StataCorp, College Station, TX, USA). All statistical tests were two-sided, where a *P* < 0.05 was considered statistically significant.

## Results

The clinical characteristics of the study population are shown in Table 1. In comparison with the patients in the control group, the HF patients were leaner and had a higher prevalence of diabetes, smoking, and CAD. Serum creatinine, fasting glucose, hemoglobin A1c (HbA1c), hsCRP, and NT-proBNP levels were all significantly higher in the HF patients. Most HF patients had impaired LVEF (mean EF, 40%). The other echocardiographic parameters, including LAd, LVEDd, and LVESd, were also greater in the HF group. In the SPECT analyses of 167 subjects, the SRS and SSS in the controls were significantly lower than those in the HF patients. This indicated a lower scar burden and CAD severity (each with a *P*< 0.0001). As expected, the proportions of the HF patients treated with diuretics, angiotensin-converting enzyme inhibitors/angiotensin receptor blockers, and β-blockers were higher than the control group.

**Table 1:** Characteristics of patients with heart failure and controls.

	Heart failure	Control	
	N = 170	N = 130	
Age (yr)	67.2 ± 14.4	66.1 ± 10.9	0.49
Male gender (%)	126 (74%)	95 (73%)	0.84
Body-mass index (kg/m^2^)	24.5 ± 4.6	25.5 ± 3.6	0.03
Waist circumference (cm)	82 ± 15	88 ± 11	0.0005
Hypertension	68 (40%)	51 (39%)	0.89
Diabetes mellitus	57 (34%)	29 (22%)	0.03
Hyperlipidemia^#^	63 (37%)	44 (34%)	0.56
Smoking	62 (36%)	33 (25%)	0.04
CAD	111 (65%)	70 (54%)	0.04
Echocardiography			
LVEF (%)	40 ± 12	69 ± 10	< 0.0001
LVEDd (mm)	56 ± 8	47 ± 5	< 0.0001
LVESd (mm)	45 ± 10	29 ± 5	< 0.0001
LAd (mm)	44 ± 8	36 ± 5	< 0.0001
Medication			
Statins	53 (31%)	38 (29%)	0.72
ACEi/ARBs	119 (70%)	41 (32%)	< 0.0001
Beta-blockers	95 (56%)	44 (34%)	0.0001
Diuretics	81 (48%)	26 (20%)	< 0.0001
Creatinine (mg/dl)	1.4 ± 0.73	0.97 ± 0.29	< 0.0001
eGFR (ml/min/1.73m^2^)	40 ± 19	50 ± 20	< 0.0001
Fasting glucose (mg/dl)	132 ± 56	113 ± 43	0.002
HbA1c (%)	7.7 ± 2.0	6.6 ± 1.5	0.004
Total cholesterol (mg/dl)	185 ± 37	192 ± 45	0.23
Triglyceride (mg/dl)*	98 (67–141)	103 (81–157)	0.07
LDL-C (mg/dl)	109 ± 33	110 ± 32	0.75
HDL-C (mg/dl)	47 ± 14	49 ± 17	0.35
hsCRP (ug/ml)*	4.73 (1.64–10.53)	1.95 (0.80–5.74)	< 0.0001
NT-proBNP (ng/L)*	1301 (504–3070)	72 (39–146)	< 0.0001
TNF-α (ng/ml)*	2.49 (1.88–3.39)	2.02 (1.47–2.51)	< 0.0001
Adiponectin (mg/L)*	10.30 (6.49–11.28)	6.71 (4.71–9.41)	0.0001
A-FABP (ng/ml)*	39.0 (17.9–49.3)	24.9 (16.2–30.9)	0.0002
ANGPTL-2 (ng/ml)*	4.63 (3.43–6.19)	3.50 (2.78–4.30)	< 0.0001

ANGPTL2, angiopoietin-like protein 2; A-FABP, adipocyte fatty acid-binding protein; ACEi/ARB, angiotensin-converting enzyme inhibitors/angiotensin receptor blockers; CAD, coronary artery disease; eGFR, estimated glomerular filtration rate; HbA1c, hemoglobin A1c; hsCRP, high-sensitivity C-reactive protein; HDL-C, high-density lipoprotein cholesterol; LDL-C, low-density lipoprotein cholesterol; LAd, left atrial dimension; LVEDd, left ventricular end-diastolic dimension; LVESd, left ventricular end-systolic dimension; LVEF, left ventricular ejection fraction; NT-proBNP, N-terminal pro-brain natriuretic peptide; TNF-α, tumor necrosis factor alpha.

* Presented with median (25^th^ to 75^th^ percentile) and analyzed by the Mann-Whitney U test

# Hyperlipidemia is defined as total cholesterol ≥ 240mg/dl, triglyceride ≥ 200mg/dl, or current use of lipid-lowering medication

The levels of adipocytokines, which include TNF-α, adiponectin, A-FABP, and ANGPTL2, were all significantly higher in the HF patients than in the controls. In the overall 300 subjects, no significant difference in ANGPTL2 concentration was observed between the sexes (men vs. women: 3.98 ng/mL [2.98–5.13 ng/mL] vs. 4.18 ng/mL [3.33–5.47 ng/mL], *P* = 0.19). The patients with diabetes had a significantly higher ANGPTL2 levels than those without diabetes (4.65ng/mL [3.64–5.86 ng/mL] vs. 3.7 ng/mL [2.93–4.96 ng/mL], *P* = 0.0002).

According to the etiology of HF, ischemic cardiomyopathy was diagnosed in 111 HF patients (65%). In addition, 135 HF patients (79%) had reduced ejection fraction. Surprisingly, the HF patients with ischemic cardiomyopathy did not have significantly higher ANGPTL2 levels than those without ischemic cardiomyopathy (4.20 ng/mL (3.15–5.65 ng/mL) vs. 3.91 ng/mL (3.00–4.98 ng/mL], *P* = 0.13). In addition, no significant differences in ANGPTL2 levels were observed between the patients with HFrEF and HFpEF (4.76 ng/mL [3.60–7.12] vs. 4.56 ng/mL [3.27–6.13 ng/mL], *P* = 0.38).

**Fig 1:**
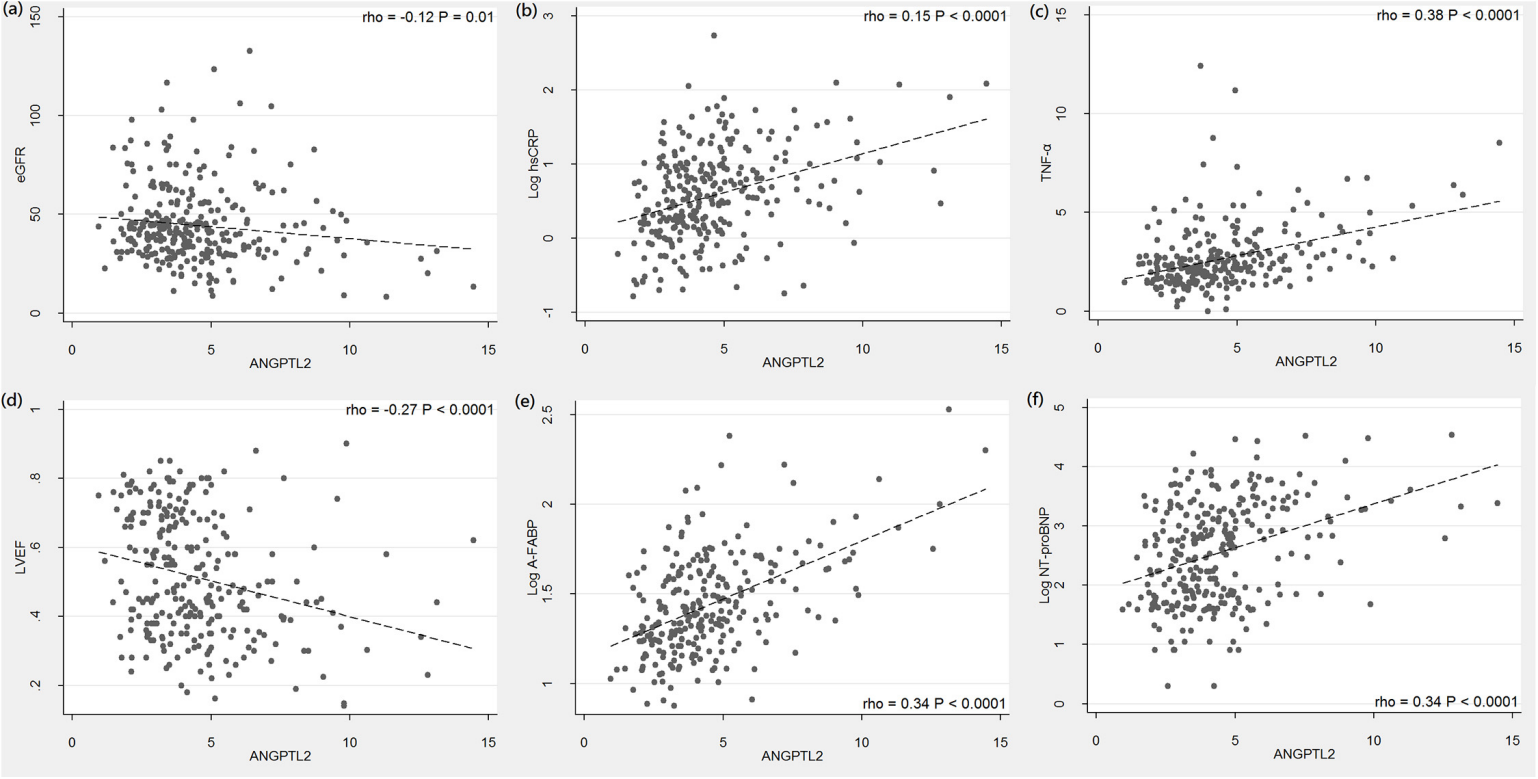
Scatter plot showing the relationship between serum ANGPTL2 levels and other clinical parameters. ANGPTL2 levels were positively correlated with hsCRP, TNF-α, A-FABP, NT-proBNP, and negatively correlated with eGFR and LVEF. (The Spearman correlation coefficients rho are presented).

Table 2 shows the results of the Spearman correlation analysis between the ANGPTL2 levels and the other clinical parameters. Several important correlation plots were demonstrated in Fig 1. In all enrolled subjects, ANGPTL2 level was positively correlated with the creatinine, fasting glucose, HbA1c, triglyceride, hsCRP, TNF-α, and A-FABP levels, and negatively correlated with HDL-C level. The NT-proBNP level, an indicator of HF severity, was also positively correlated with ANGPTL2 level (rho = 0.34, *P*< 0.0001; Fig 1F). Among the echocardiographically derived parameters, ANGPTL2 level inversely correlated with LVEF (rho = −0.27, *P*< 0.0001; Fig 1D) but positively correlated with LVEDd, LVESd, and LAd. In order to determine which variables independently correlated with ANGPTL2 level, parameters that were significantly associated with ANGPTL2 level were included in a further multiple stepwise regression analysis. This revealed that HDL-C, A-FABP, and hsCRP levels were independently associated with ANGPTL2 level (Table 3).

**Table 2:** Spearman correlation of ANGPTL2 levels with cardiovascular risk factors.

	Univariate	
	rho	P value
Age	0.08	0.19
Body-mass index	0.04	0.55
Waist circumference	-0.12	0.05
Creatinine	0.32	0.003
Fasting glucose	0.18	0.004
HbA1c	0.26	0.01
Total cholesterol	-0.08	0.20
Triglyceride	0.13	0.04
HDL-C	-0.29	< 0.0001
LDL-C	0.002	0.97
hsCRP	0.40	< 0.0001
NT-proBNP	0.34	< 0.0001
TNF-α	0.38	< 0.0001
A-FABP	0.34	< 0.0001
Adiponectin	-0.04	0.59
**Echocardiography**		
LVEF	-0.27	< 0.0001
LVEDd	0.13	0.03
LVESd	0.22	0.0003
LAd	0.34	< 0.0001

**Table 3:** Multiple stepwise regression analysis showing the variables independently associated with the serum level of ANGPTL2.

Parameters	β	S.E.	Standardized β	P value
hsCRP	0.63	0.21	0.23	0.004
A-FABP	0.16	0.45	0.27	0.001
HDL-C	-0.003	0.0008	-0.30	< 0.001

Variables included in the original model are age, gender, fasting glucose, HbA1c, triglyceride, HDL-C, creatinine, hsCRP, TNF-α, A-FABP, and NT-proBNP

* ANGPTL2, triglyceride, hsCRP, A-FABP, TNF-α, and NT-proBNP are logarithmically transformed before analysis.

**Fig 2:**
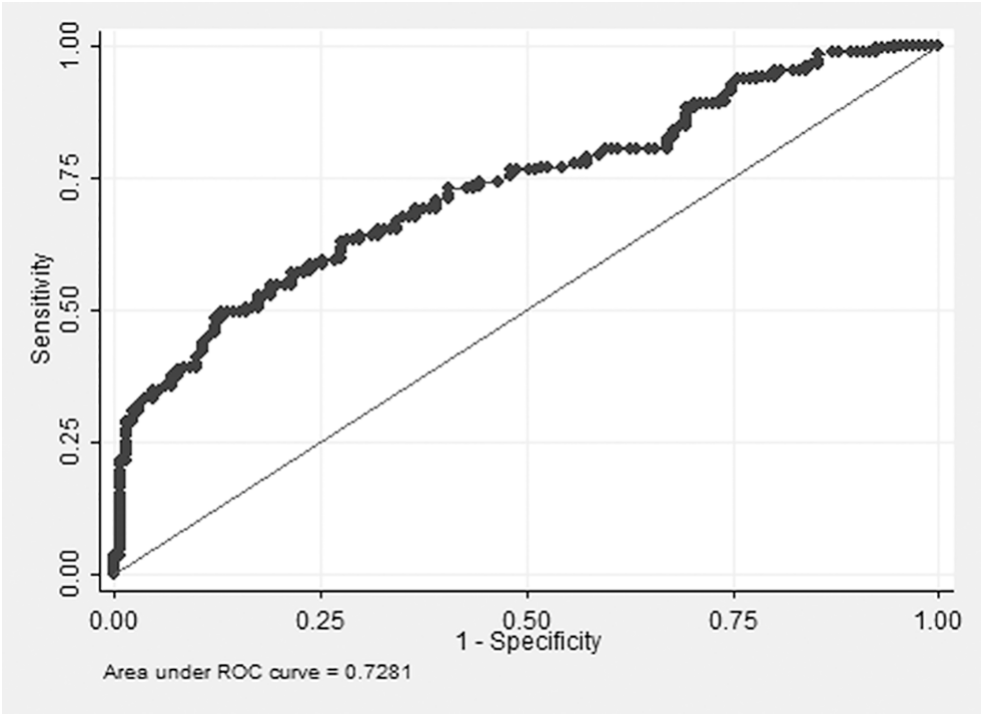
The receiver operating characteristic (ROC) curve of serum ANGPTL2 levels for the identification of patients with heart failure.

A receiver operating characteristic (ROC) curve was generated using the different ANGPTL2 cut-offs for the screening of the HF. The areas under the ROC curve, 95% confidence intervals, and the point to the ROC curve closest to 1, were calculated. The area under the ROC curve was 0.73 (95% confidence interval [CI], 0.68–0.77) with the best cut-off level 4.12 ng/mL (Fig 2). In order to evaluate the relationship between circulating ANGPTL2 level and HF, the circulating ANGPTL2 levels were divided into tertiles (ANGPTL2 level ≤3.4 ng/mL, n = 100; 3.4 ng/mL< ANGPTL2 level ≤ 4.8 ng/mL, n = 100; ANGPTL2 level >4.8 ng/mL, n = 100) and analyzed as categorical variables. A multivariate logistic regression analysis was performed by using the HF presence as a dependent variable (Table 4). For the subjects with ANGPTL2 levels in the highest tertile, their risk of HF was about 5.88-fold (95% confidence interval [CI], 3.10–11.16) higher than those with ANGPTL2 levels in the lowest tertile. After adjusting the potential confounding factors, the odds ratio remained statistically significant (2.97; 95% CI, 1.24–7.08; *P* value for trend = 0.02).

**Table 4:** Multivariate logistic regression analyses showing the odds ratios (OR) for the presence of heart failure in different levels of circulating ANGPTL2.

	n	Model 1			Model 2		
ANGPTL2		OR	95% CI	P value	OR	95% CI	P value
Lowest tertile	100	1.0			1.0		
Middle tertile	100	1.21	0.69–2.13	0.51	1.15	0.54–2.47	0.72
Highest tertile	100	5.88	3.10–11.16	< 0.001	2.97	1.24–7.08	0.01
P for trend				< 0.001			0.02

Lowest tertile: ANGPTL2 level ≤3.4 ng/mL; middle tertile: 3.4 ng/mL< ANGPTL2 level ≤ 4.8 ng/mL; highest tertile: ANGPTL2 level >4.8 ng/mL

Model 1 adjusted for age and gender

Model 2 adjusted for age, gender, body-mass index, diabetes, hyperlipidemia, fasting glucose, creatinine, hsCRP, TNF-α, adiponectin, NT-proBNP, and diagnosis of CAD

## Discussion

The major finding in the present study was that serum ANGPTL2 level, which was significantly higher in the HF patients than those without HF, was an independent predictor of the presence of HF in the multivariate analysis. Moreover, ANGPTL2 level was not only associated with inflammatory and metabolic parameters but also positively correlated with the level of NT-proBNP, a well-documented marker of HF severity.

ANGPTL2 is abundantly expressed in the heart, adipose tissues, lungs, kidneys, and skeletal muscles [21]. Among these tissues, visceral fat is believed to have the greatest contribution to circulating glycoprotein level [9]. ANGPTL2 expression is stimulated by hypoxia, and induces both angiogenesis and endothelial cell migration [21]. ANGPTL2 expression has recently been associated with rheumatoid arthritis, dermatomyositis, cancer, abdominal aortic aneurysms, and CAD [13,22–25]. Data have suggested that ANGPTL2 expression also promotes inflammation and contributes to atherosclerotic pathogenesis [13]. In the animal model, ANGPTL2 deletion ameliorated both the adipose tissue inflammation and systemic insulin resistance in diet-induced obese mice [9]. Conversely, ANGPTL2 overexpression in adipose tissue also caused local inflammation and systemic insulin resistance in non-obese mice [9]. In humans, ANGPTL2 concentration is upregulated in obesity and correlated with both systemic insulin resistance and inflammation levels [7]. The positive correlation between ANGPL2, fasting glucose, HbA1c, hsCRP, and TNF-α in our current study supported these findings as well (Table 2). Obesity have been demonstrated to induce adipose tissue inflammation, which further promote ANGPTL2 overexpression [7]. Because HF also induces adipose tissue inflammation, the same mechanism might possibly cause the ANGPTL2 upregulation in patients with HF. The upregulation of adiponectin levels in our HF group patients were presented in previous studies as well, and it is believed to be a compensatory mechanism to the development of adiponectin resistance [26]. Several studies have demonstrated that A-FABP, another important adipocytokine, is involved in the development of insulin resistance, inflammation and atherosclerosis, and the circulating A-FABP level is upregulated in patients with HF as well [27,28]. The multiple regression results revealed that A-FABP was also an independent predictor for ANGPTL2 (Table 3). Because the evidence that ANGPTL2 has a direct effect on lipid metabolism is unclear, the positive correlation with triglyceride and the negative correlation with HDL-C level might reflect the HF-associated metabolic derangements.

Various components of metabolic syndrome and obesity have been associated with an increased risk of developing atherosclerosis, cardiac hypertrophy, ischemic heart disease, and ultimately HF [29]. Meanwhile, HF patients also had identifiable metabolic derangements and dysregulation of several adipocytokines. These factors are not only associated with HF severity but also predict cardiovascular outcomes [30–32]. Several studies not only have shown the correlation between adipocytokines and atrial fibrillation but also provided evidence that adipocytokines directly affect glucose transport, contractile function, and hypertrophy in cardiomyocytes [33–35]. In our present study, echocardiographically derived LV function was negatively correlated with ANGPTL2 levels **(** Table 2 **)**. However, the pathophysiological effects of ANGPTL2 expression on cardiomyocytes remain deficient and deserve further comprehensive study.

Unexpectedly, we did not find a significant difference in circulating ANGPTL2 levels between the patients with and those without CAD (CAD vs. non-CAD: 4.12 ng/mL [3.15–5.65 ng/mL] vs. 3.91 ng/mL [3–4.98 ng/mL], *P* = 0.23). Considering that coronary angiography was not performed in each subject, some misclassification of CAD could have existed in our study. However, taking SSS as an indicator of both CAD and its severity, ANGPTL2 levels were higher in the patients with severely abnormal SSS than in those with normal SSS (SSS > 12 vs. SSS ≤ 4: 4.21 ng/mL [3.32–4.93 ng/mL] vs. 3.43 ng/mL [2.81–4.25 ng/mL], *P* = 0.03). Even so, the ANGPTL2 levels in the HF patients with normal myocardial perfusion study results (n = 30) remained significantly higher than those in the control subjects (4.36 ng/mL [3.50–5.30 ng/mL] vs. 3.50 ng/mL [2.78–4.30 ng/mL], *P* = 0.02). That is, the upregulated circulating ANGPTL2 levels in the HF patients could not simply be explained by the presence of advanced atherosclerosis.

Notably, higher serum creatinine levels and lower eGFR are observed in HF patients. The impaired renal function might induce HF symptom developments. However, ANGPTL2 may not be freely filtrated in the normal glomerulus because of its 57 kDa molecular weight. The negative correlation between eGFR and ANGPTL2 was also reported in previous studies, but creatinine was not independently associated with ANGPTL2 in multiple regression analysis [10,36]. In addition, we found a borderline negative correlation between ANGPTL2 and waist circumference in overall study population (rho = -0.12, P = 0.05; Table 2), especially in patients with HF (rho = -0.17, P = 0.03). On the other hand, HF group patients had significantly lower waist circumference (82cm vs. 88cm, P = 0.0005), which might be associated with cardiac cachexia. Probably that the severity of adipose tissue inflammation, but not the adipose tissue volume, is the main determinant of circulating ANGPTL2 level in patients with HF.

This study has some limitations. First, women represented only 26% of the HF patients. In addition, only 21% of the HF patients were diagnosed with HFpEF; the patient number or percentage was far lower than that reported in other studies published in the literature. Second, body fat component and insulin level data were not collected in the present study. Therefore, the relationship between circulating ANGPTL2 levels, visceral fat, and insulin resistance index could not be analyzed. Third, the New York Heart Association (NYHA) Functional Classes were not detailed recorded in lots of HF patients. Therefore, the relationship between ANGPTL2 level and NYHA Functional Classification could not be evaluated in our current study. Finally, our current study design was cross-sectional, precluding the analysis of a cause-and-effect correlation between ANGPTL2 level and HF. In fact, in a short-term follow-up (median 43 months) of HF group patients, we failed to demonstrate any significant role of serum ANGPTL2 level in cardiovascular outcomes prediction.

## Conclusions

This is the first study to demonstrate an independent correlation between serum ANGPTL2 level and HF. Our results also provided evidence of a close relationship between ANGPTL2 level, inflammation, and metabolic abnormalities. Hence, ANGPTL2 level should be considered as a novel HF biomarker. Further prospective study design is warranted to evaluate the associations of ANGPTL2 concentrations with HF incidence and cardiovascular outcomes.
